# Development and Validation of a Panel of One-Step Four-Plex qPCR/RT-qPCR Assays for Simultaneous Detection of SARS-CoV-2 and Other Pathogens Associated with Canine Infectious Respiratory Disease Complex

**DOI:** 10.3390/v15091881

**Published:** 2023-09-05

**Authors:** Côme J. Thieulent, Mariano Carossino, Laura Peak, Keith Strother, Wendy Wolfson, Udeni B. R. Balasuriya

**Affiliations:** 1 Louisiana Animal Disease Diagnostic Laboratory, School of Veterinary Medicine, Louisiana State University, Baton Rouge, LA 70803, USA; cthieulent@lsu.edu (C.J.T.); mcarossino1@lsu.edu (M.C.); lpeak1@lsu.edu (L.P.); kstrother@lsu.edu (K.S.); 2 Department of Pathobiological Sciences, School of Veterinary Medicine, Louisiana State University, Baton Rouge, LA 70803, USA; 3 Department of Veterinary Clinical Sciences, School of Veterinary Medicine, Louisiana State University, Baton Rouge, LA 70803, USA; wwolson@lsu.edu

**Keywords:** multiplex reverse-transcriptase qPCR (RT-qPCR), canine respiratory pathogens, canine infectious respiratory disease complex (CIRDC), SARS-CoV-2, influenza virus A

## Abstract

Canine infectious respiratory disease complex (CIRDC) is the primary cause of respiratory disease in the canine population and is caused by a wide array of viruses and bacterial pathogens with coinfections being common. Since its recognition in late 2019, Severe Acute Respiratory Syndrome Coronavirus-2 (SARS-CoV-2) has been reported to cause respiratory disease in dogs. Therefore, the rapid detection and differentiation of SARS-CoV-2 from other common viral and bacterial agents is critical from a public health standpoint. Here, we developed and validated a panel of four one-step multiplex qPCR/RT-qPCR assays for the detection and identification of twelve pathogens associated with CIRDC (canine adenovirus-2, canine distemper virus, canine herpesvirus-1, canine influenza A virus, canine parainfluenza virus, canine pneumovirus, canine respiratory coronavirus, SARS-CoV-2, *Bordetella bronchiseptica*, *Streptococcus equi* subsp. *zooepidemicus*, *Mycoplasma cynos,* and *M. canis*), as well as the identification of three main CIV subtypes (i.e., H3N2, H3N8, and H1N1). All developed assays demonstrated high specificity and analytical sensitivity. This panel was used to test clinical specimens (*n* = 76) from CIRDC-suspected dogs. *M. canis*, *M. cynos*, and CRCoV were the most frequently identified pathogens (30.3%, 25.0%, and 19.7% of samples, respectively). The newly emerging pathogens CPnV and SARS-CoV-2 were detected in 5.3% of samples and coinfections were identified in 30.3%. This new multiplex qPCR/RT-qPCR panel is the most comprehensive panel developed thus far for identifying CIRDC pathogens, along with SARS-CoV-2.

## 1. Introduction

Canine infectious respiratory disease complex (CIRDC) is a contagious disease syndrome, commonly referred to as “kennel cough” or “canine cough”, which is caused by a wide array of etiologic agents [1,2]. Outbreaks of CIRDC have been reported all around the globe [3,4,5,6,7,8] and most commonly occur when dogs are housed or concentrated together, such as in boarding facilities, animal shelters, dog daycare, racing facilities, and obedience training classes [3,9]. Cases of CIRDC have also been reported in veterinary hospitals [10] and individually owned dogs [3]. Common clinical signs include coughing, nasal or ocular discharge, sneezing, and respiratory distress that typically lasts one to two weeks [2]. This multifactorial disease complex is associated with several pathogens, including viruses and bacteria [2,11]. The pathogens traditionally associated with CIRDC include canine adenovirus-2 (CAdV-2), canine distemper virus (CDV), canine herpesvirus-1 (CHV-1), canine parainfluenza virus (CpiV), and *Bordetella bronchiseptica* [2,12,13,14,15,16]. Since the early 2000s, it has been demonstrated that other pathogens are also involved in CIRDC, including Canine Respiratory Coronavirus (CRCoV), *Streptococcus equi* subsp. *zooepidemicus*, *Mycoplasma* spp., and canine influenza A virus (CIV). CRCoV was first detected in 2003 in dogs with respiratory distress [17] and is now well-recognized to be associated with CIRDC [18]. *Streptococcus equi* subsp. *zooepidemicus* is highly contagious and associated with severe cases of respiratory disease [19,20]. *Mycoplasma* spp. are part of the bacteria flora of the dog’s upper respiratory airways; among the 15 known species of *Mycoplasma* reported in dogs, only *M. cynos* and *M. canis* were associated with respiratory disorders [12,21,22,23]. While the pathogenic role of *M. cynos* was recently supported by meta-analyses, the role of *M. canis* was not. [24]. Among CIV subtypes, H3N8 and H3N2 are the most prevalent in the canine population around the world [25]. CIV H3N8, derived from equine influenza A virus H3N8, was first identified in 2004 in Florida, USA [26]. CIV H3N2, suspected to be derived from an avian influenza A virus in China and Korea [27], was first detected in the USA in a 2015 CIRDC outbreak in Chicago, Illinois [28]. Moreover, following the 2009 H1N1 human pandemic, cases of H1N1 infection in dogs have also been documented [25,29,30]. Canine pneumovirus (CPnV) is an emerging pathogen first isolated in the USA in 2010 from a dog with respiratory disease [31,32]. Since then, CPnV has been reported in dogs with CIRDC in North America, Europe, and Asia [3,4,8,33,34,35,36,37]. Since the causative agents of CIRDC induce similar clinical signs, confirmatory diagnosis relies on laboratory testing. Additionally, co-infections by two or more viral and/or bacterial pathogens is commonly observed and can lead to more severe clinical signs when compared to single infections [8,12,38].

Numerous reports have emerged during the COVID-19 pandemic of SARS-CoV-2 transmission from humans to a number of farm, wild, zoo, and companion animals, including dogs and cats, during the COVID-19 pandemic [39,40,41]. Among companion animals (i.e., dogs and cats), SARS-CoV-2 infections can either be subclinical or associated with respiratory (e.g., nasal discharge, coughing, dyspnea) and/or gastrointestinal (e.g., vomiting and diarrhea) clinical signs [42,43,44,45,46]. Nevertheless, cases of SARS-CoV-2 infection in dogs typically result in none to mild clinical manifestations and are not considered significant contributors to the spread of the virus [47]. While studies conducted on dogs experimentally infected with SARS-CoV-2 have revealed that they do not display signs of illness [44,48,49], transmission from experimentally infected dogs to naive dogs (sentinel controls) has been documented [50]. This raises concerns about the possible spillover between humans and dogs, requiring continuous surveillance to monitor SARS-CoV-2 infection in companion animals. Furthermore, the clinical signs induced by SARS-CoV-2 are not unique; thus, veterinarians must rule out more common causes of respiratory in animals by seeking laboratory diagnosis. Thus, rapid detection and differentiation of SARS-CoV-2 from other common viral and bacterial agents is critical for controlling COVID-19 and implementing appropriate biosecurity measures to prevent transmission.

Most of the veterinary diagnostic laboratories offer singleplex real-time PCR (qPCR) and reverse transcriptase-qPCR (RT-qPCR) assays to detect various respiratory pathogens (viral and bacterial) in clinical specimens of animals. However, using multiplex assays for the identification of two or more targets in a single reaction has several advantages. Firstly, a multiplex qPCR and RT-qPCR reduces the amount of valuable clinical samples needed for testing. Secondly, multiplexing reduces the cost by amplifying two or more targets (up to four) in one well, saving reagents and the technical time and effort needed to set up the tests and analyze the results. Finally, the amplification of multiple genes in the same well improves precision by minimizing pipetting errors. One of the limitations of multiplexing is related to the overlapping emission and spectra that some florescent dyes have, which limits their use in multiplex scenarios. This issue has been overcome in recent years with a new generation of fluorescent dyes by several manufacturers. [51,52,53,54].

Thus, in this study, we developed and evaluated the performance of a panel of four four-plex one-step qPCR/RT-qPCR assays for the simultaneous detection and differentiation of pathogens associated with CIRDC (e.g., viruses and bacteria) and SARS-CoV-2, namely canine respiratory assay 1 (CRA_1), CRA_2, and CRA_3, as well as an RT-qPCR assay to differentiate CIV H3N2, H3N8, and H1N1subtypes (CRA_4). This new panel was then used to test clinical samples submitted to the Louisiana Animal Disease Diagnostic Laboratory (LADDL) from CIRDC-suspected dogs collected in Louisiana, USA, between 2020 and 2023. Overall, this new highly sensitive and specific panel of multiplex qPCR/RT-qPCR assays developed in this study can simultaneously detect all CIRDC pathogens and SARS-CoV-2 in respiratory specimens.

## 2. Materials and Methods

### 2.1. Viruses and Bacteria

A panel of reference (prototype) viruses and bacteria associated with CIRDC and genetically related pathogens was used to assess the specificity (inclusivity/exclusivity) of each assay in singleplex and multiplex format (Table 1). RNA of CIV H3N2 VSL-1355 and CRCoV VSL-1471 were kindly provided by Dr. Diego Diel (Department of Population Medicine and Diagnostic Sciences, Cornell University College of Veterinary Medicine, Ithaca, NY, USA). RNA of CIV H3N8 A/Ca/FL/15592/04 and A/Ca/FL/61156.2/07 were kindly provided by Dr. Edward Dubovi. All other prototype strains were obtained from the American Type Culture Collection (ATCC^®^; Manassas, VA, USA) or BEI Resources (Manassas, VA, USA).

### 2.2. Clinical Specimens

A total of 76 specimens including nasal swabs (*n* = 38), pharyngeal swabs (*n* = 29), and pools of tissues (*n* = 2) submitted for routine diagnostic testing at the Louisiana Animal Disease Diagnostic Laboratory (LADDL) between 2020 and 2023 were included in this study. These specimens were collected from a total of 50 dogs, with nasal swabs and pharyngeal swabs concurrently collected in 26/50 dogs (Table S1). Swabs were either submitted to the LADDL for CIRDC diagnosis or collected from three shelters located in and around Baton Rouge, LA, USA. Specimens were collected using sterile oropharyngeal/nasal swabs (VMRD, Pullman, WA) and all swab samples were resuspended in either 2 mL of BHI Broth (Hardy Diagnostics, Santa Maria, CA, USA) or 2 mL of PrimeStore^®^ molecular transport medium (VMRD). Swab samples were then vortexed and centrifuged for clarification and the supernatant was stored at 4 °C until use. Tissues (i.e., kidney, liver, lung, spleen) were collected from dogs submitted for necropsy at the LADDL and were homogenized using the Bead Ruptor Elite (Omni, Inc, Dallas, TX, USA) in a 1:9 ratio with 1× phosphate-buffered saline (PBS) by performing two cycles of 30 s at 4.00 m/s. Samples were then clarified by centrifugation at 4000× *g* for 10 min at 4 °C.

### 2.3. Nucleic Acid Extraction

Nucleic acid extraction was performed using the taco^TM^ mini nucleic acid automatic extraction system (GeneReach, Taichung, Taiwan) following manufacturer’s recommendations. One hundred microliters of swab or 10% tissue suspensions were extracted and eluted in equal volume of elution buffer. The extracted nucleic acid samples were stored at −80 °C until used.

### 2.4. Primers and Probe Design

Specific forward and reverse primers and probes used for specific amplification of CPiV nucleocapsid (N), CDV phosphoprotein (P), CPnV-N, CRCoV-N, *M. canis* tuf gene, CIV H3N2 neuraminidase (NA), and CIV H1N1-NA assays were designed using Geneious R6 software (v.6.1.8, Auckland, New Zealand) and IDT’s PrimerQuest tool (https://www.idtdna.com/Primerquest/home/Index, accessed on 1 September 2022) from sequences available on the GenBank nucleotide database (https://www.ncbi.nlm.nih.gov/nuccore/, accessed on 1 September 2022) and Influenza Research Database (https://www.fludb.org, accessed on 1 September 2022) (Table 2). The primers and probe sequences specificity were further validated in silico using the NCBI Basic Local Alignment Search Tool (BLAST; https://blast.ncbi.nlm.nih.gov/Blast.cgi?PROGRAM=blastn&PAGE_TYPE=BlastSearch&LINK_LOC=blasthome, accessed on 1 September 2022). Self-annealing sites, hairpin loop formation, and 3’ complementarity were analyzed using IDT’s OligoAnalyzer tool (https://www.idtdna.com/calc/analyzer, accessed on 1 September 2022). Sequences of primers and probes for SARS-CoV-2 [55], CHV-1 [56], *Bordetella bronchiseptica* [57], *M. cynos* [58], *Streptococcus equi* subsp. *Zooepidemicus* [59], and CIV H3N8 [60,61] detection were used as previously published (Table 2). CIV [62] and CAdV-2 [63] primers and probes were used as previously published with addition of nucleotide degeneracy in the sequences of the CIV reverse primer 1 (CIV_M-R1 position 1: T→Y) and CAdV-2 probe (CAdV2_H-P position 10: T→Y), respectively (Table 2).

### 2.5. Specific Multiplex TaqMan^®^ Quantitative PCR (qPCR) and Reverse Transcription PCR (RT-qPCR) Assays for Canine Respiratory Pathogens

Four four-plex RT-qPCR assays were developed and designated as follows: CRA_1 (detection of CIV, CDV, CpiV, and CAdV-2), CRA_2 (detection of CRCoV, CPnV, CHV-1, and SARS-CoV-2), CRA_3 (detection of *B. bronchiseptica*, *S. equi* subsp. *zooepidemicus*, *M. cynos,* and *M. canis*) and CIV_4 for identification of CIV and its most prevalent subtypes in dogs (i.e., CIV-H3N2, CIV-H3N8, CIV-H1N1). RT-qPCR assays were performed in a total volume of 25 μL containing 12.5 μL of 2× QuantiTect^TM^ Multiplex RT-PCR Master Mix (QIAGEN, Hilden, Germany), 0.25 μL of QuantiTect^TM^ RT Mix, 1.25 μL of primers and fluorogenic probes mix (200 nM each), 6 μL of RNase free water, and 5 μL of template DNA/RNA. A 7500 Fast Real-Time PCR System (Applied Biosystems, Waltham, MA, USA) was used with the following thermal profile: a reverse transcription step (20 min at 50 °C) followed by an initial activation step (15 min of at 95 °C) and 40 cycles of denaturation and annealing/extension (45 s at 94 °C and 75 s at 60 °C). The complete step-by-step protocols have been deposited on protocol.io platform (DOIs: dx.doi.org/10.17504/protocols.io.kxygx9x7zg8j/v1; dx.doi.org/10.17504/protocols.io.14egn2o9pg5d/v1).

### 2.6. Synthesis of In Vitro Transcribed RNA and DNA

Specific in vitro transcribed (*IVT*) RNA and plasmid DNA were synthesized in order to determine the analytical sensitivity of each multiplex RT-qPCR assay as previously described, with minor modifications [53]. Four inserts containing the target regions of each assay flanked by *PstI* and *HindIII* restriction enzymes were chemically synthesized and cloned into the pGEM^®^-3Z vector (Promega, Madison, WI, USA) downstream of the T7 promoter by GeneArt Gene Synthesis (Thermo Fisher Scientific, Waltham, MA, USA). Transformed Escherichia coli DH10β cells were cultured overnight at 37 °C with agitation at 270 rpm. Plasmid DNA was extracted using QIAprep Spin Miniprep kit (QIAGEN). Plasmid DNA was linearized using HindIII restriction enzyme and concentration was measured using Qubit dsDNA BR Assay Kit (Thermo Fisher Scientific). When *IVT* RNA was needed, linearized plasmid DNA were subjected to in vitro transcription using the Megascript^®^ T7 Transcription Kit (Thermo Fisher Scientific) following manufacturer’s recommendations. Subsequently, DNase treatment was performed with TURBO^TM^ DNase (Thermo Fisher Scientific) for 15 min at 37 °C. The *IVT* RNA products were purified using MEGAclear^TM^ Transcription Clean-Up Kit (Thermo Fisher Scientific) and quantified using Qubit RNA BR Assay Kit (Thermo Fisher Scientific). The number of plasmid DNA and *IVT* RNA (copies/μL) were calculated according to the following formula:

$$\mathrm{Number}\;\mathrm{of}\;\mathrm{plasmid}\;\mathrm{DNA}/\mathrm{IVT}\;\mathrm{RNA}\;\mathrm{molecules}/\mu L=\frac{Avogadro^{\prime }s\;number\;\left(6.022\times 10^{23}\right)\times plamid\;DNA/IVTRNA\;concentration\;\left(\frac{g}{\mu L}\right)}{plasmid\;DNA/IVT\;RNA\;molecular\;weight\;\left(\frac{g}{\mathrm{mol}}\right)}$$

*IVT* RNA and DNA plasmid molecular weight was calculated using Molbiotools website (https://molbiotools.com/dnacalculator.php, accessed on 1 October 2022). Each *IVT* RNA and plasmid DNA concentration was adjusted to 10^7^ copies/μL in nuclease-free water and stored at −80 °C until used. Then, 40 ng/μL of yeast tRNA (Thermo Fisher Scientific) was added in *IVT* RNA preparations. Ten-fold serial dilutions of *IVT* RNA and plasmid DNA were directly used for determining the analytical sensitivity of the qPCR assays.

### 2.7. Analytical Parameter Determination and Statistical Analysis

Analytical parameters were determined as previously described [53], with minor modifications. Standard curves were generated using ten-fold dilutions of plasmid DNA and *IVT* RNA (10^7^ to 10^1^ copies/μL) in triplicate. Coefficients of determination (R^2^) were used to assess curve fitness. Amplification efficiency [E (%)] was calculated after regression analysis using the following formula: E = [10^−1/slope^ − 1] × 100. Limit of detection with 95% confidence (LOD_95%_) of each assay was determined by statistical probit analysis (non-linear regression model) using SPSS 14.0 software (SPSS Inc., Chicago, IL, USA) from twelve replicates per dilution ranging from 10^3^ to 10^0^ copies/μL. Cycle threshold (Ct) cut-off values were determined using the following formula: Ct cut-off = Average Ct values of 12 replicates of the endpoint dilution + (3 × standard deviation [SD]) [53,54,65]. Intra-run imprecision was determined by performing 12 replicates of plasmid DNA/*IVT* RNA containing 10^5^ to 10^3^ copies/μL on the same run and inter-run imprecision was determined by using three replicates of plasmid DNA/*IVT* RNA containing 10^5^ to and 10^3^ copies/μl on two independent runs. The coefficient of variation (%CV) was calculated using the following formula: %CV = 100 × (standard deviation of replicates [log_10_ copies/µL] ÷ average of replicates [log_10_ copies/µL]). Data were graphically represented using GraphPad Prism v9.3.1 statistical analysis software (GraphPad, San Diego, CA, USA) and UpSetR package [66].

## 3. Results

### 3.1. Analytical Specificity of Singleplex and Multiplex Assays for the Detection of Canine Respiratory Pathogens

The analytical specificity (inclusivity/exclusivity) of all singleplex and four-plex qPCR/RT-qPCR assays was first evaluated using a panel of reference viruses and bacteria associated with respiratory disease in dogs, unrelated pathogens (i.e., CAdV-1, Canine enteric coronavirus [CECoV], and *M. felis*), and different SARS-CoV-2 variants of concern (VOCs). The previously published assays [55,56,57,58,59,60,61,62,63] and the news assays developed in this study showed exclusive specificity for the respective targets and did not cross-react between each other when used under multiplex conditions (Figure S1). As no prototype CPnV strain was available, the closely related murine pneumonia virus strain 15 (ATCC^®^, VR-1819^TM^) was used [67] and detected by the newly developed CPnV (N) assay. The CAdV-2 (H) assay did not amplify the CAdV-1 reference strain, the CRCoV (N) assay did not amplify the CECoV reference strain, the *M. cynos* (tuf) assay did not amplify the *M. canis* and *M. felis* reference strains, and the *M. canis* (tuf) assay did not amplify the *M. cynos* and *M. felis* reference strains.

### 3.2. Analytical Sensitivity of Singleplex and Multiplex Assays for the Detection of Canine Respiratory Pathogens

**Figure 1:**
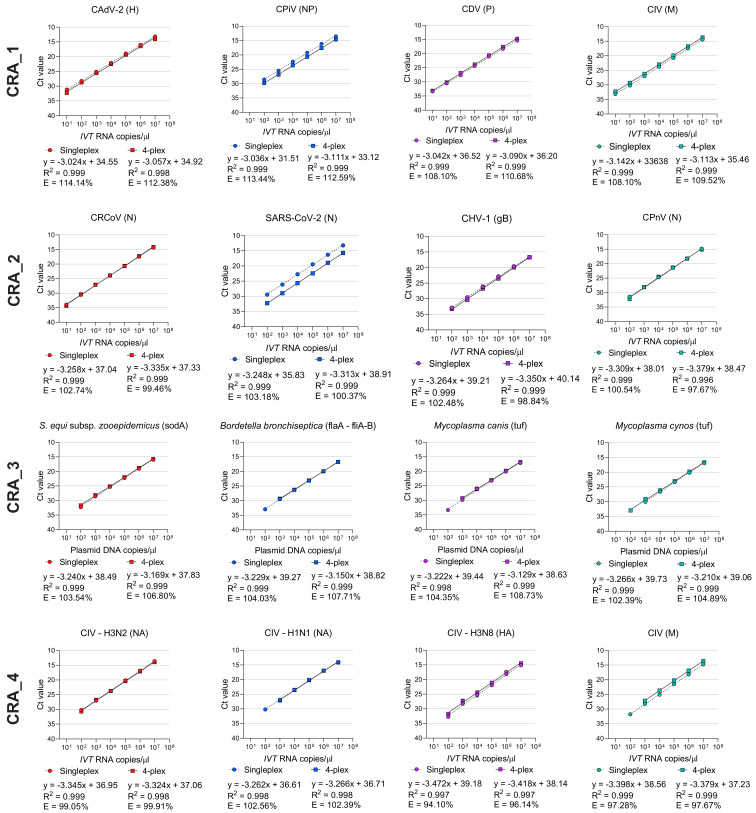
Comparison of analytical sensitivity of each singleplex and multiplex qPCR and RT-qPCR assays for the detection of pathogens associated with CIRDC and SARS-CoV-2. Ct: cycle threshold; *IVT* RNA: in vitro transcribed RNA; R^2^: linearity; E: efficiency.

The analytical sensitivity of all assays in singleplex and in multiplex were determined using ten-fold dilutions (10^7^ copies/µL to 10^2^ copies/µL) of the specific plasmid DNA/IVT RNA containing the target sequences. Linear standards curves were generated for each assay in singleplex and four-plex with a coefficient of linear regression (R^2^) ≥ 0.997 (Figure 1 and Table 3). The amplification efficiency for each singleplex and four-plex assay was similar and ranged between 94.10% and 114.35% (Table 3).

The detection rate limit (100%) was equal to 10–100 copies/µL for all assays when ran in singleplex and in four-plex conditions. The limit of detection (LOD_95%_) calculated using a probit analysis was ≤15 copies/µL for all singleplex assays, except for *M. canis* (tuf) and *M. cynos* (tuf) where the LOD_95%_ was slightly higher with values of 28 and 43 copies/µL, respectively (Table 3 and Figure S2). A similar LOD_95%_ was observed for most of these assays when performed in a multiplex format. An increase in the LOD_95%_ was noted for *B. bronchiseptica* (flaA-fliA-B) but was less than 1 Log_10_.

### 3.3. Repeatability and Reproducibility of Multiplex qPCR/RT-qPCR Assays for Detection of Canine Respiratory Pathogens

The repeatability and reproducibility of each four-plex assay was determined by measuring the intra-run and inter-run variability, respectively. Three concentrations of plasmid DNA/IVT RNA were used: 10^5^ copies/µL (high concentration), 10^4^ copies/µL (medium concentration), and 10^3^ copies/µL (low concentration). The coefficients of variability (CV) are presented in Table 4. For all assays, the intra-run variability was <2% at high concentration, <4% at medium concentration, and <6% at low concentration. Similarly, the inter-run variability was <2% at high concentration, <3% at medium concentration, and < 5% at low concentration.

### 3.4. Use of Multiplex Assays on Biological Specimens from CIRDC-Suspected Dogs

**Figure 2:**
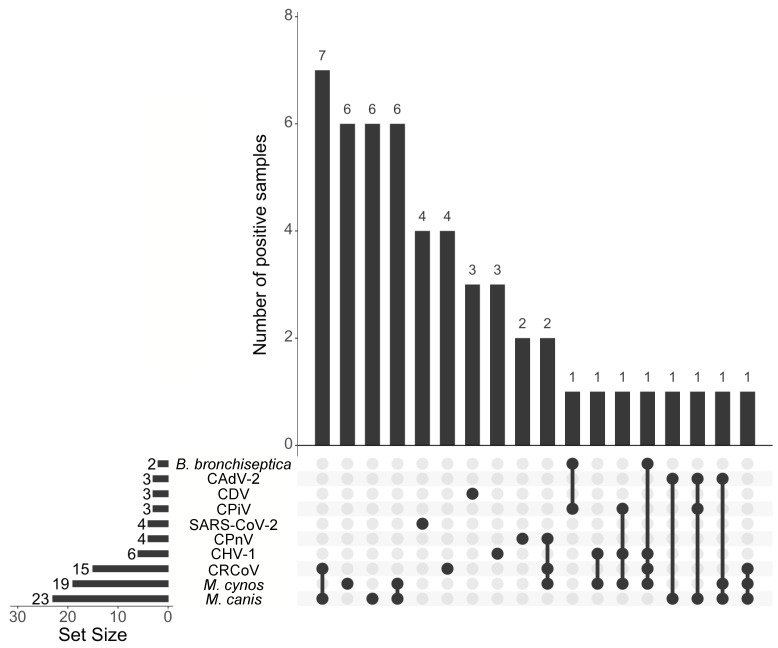
UpSet plot summarizing the number of CIRDC pathogens and SARS-CoV-2 detected in dogs using the newly developed panel. The number samples with single infection or co-infection are shown as vertical bars. The bottom left horizontal bar graph labeled Set Size shows the total number of positive samples for each specific CIRDC pathogens and SARS-CoV-2.

The panel of multiplex assays was used to test 76 clinical samples collected from dogs that displayed respiratory disease between 2020 and 2023 in Louisiana, USA. Among the 76 samples, 51 (67.1%) were positive for at least one of the 12 pathogens tested, *M. canis* (*n* = 23; 30.3%), *M. cynos* (*n* = 19; 25.0%), and CRCoV (*n* = 15; 19.7%) were the most commonly identified pathogens (Figure 2; Table S2). No samples were positive for *S. equi* subsp. *zooepidemicus* or CIV. SARS-CoV-2 was detected in four samples (5.3%) collected in August 2021. The first SARS-CoV-2-positive sample was a nasal swab collected from a 4-year-old female Goldendoodle presenting with leukocytosis, fever, and lethargy. The second SARS-CoV-2 positive sample was a pharyngeal swab collected from a six-year-old male Cocker Spaniel with a cough. The respective owners of these dogs have previously tested positive for COVID-19. No record of the dogs’ clinical signs and owner status was available for the two other samples collected from a 7-year-old female Schnauzer and a one-year-old female Goldendoodle. Additionally, four samples were positive for CPnV RNA (5.3%); these samples were collected from two dogs located in the same shelter (nasal and pharyngeal swabs).

Co-infections were identified in 23 samples (30.3%) and ranged from two (16 samples, 21.1%) to up to four agents (one sample, 1.3%) (Figure 2; Table S3). *Mycoplasma* species were typically the most common co-infecting agent.

## 4. Discussion

CIRDC is a complex infectious disease in dogs caused by one or a combination of several viruses and bacteria [2]. A rapid detection of the implicated pathogen(s) is important in order to provide the most appropriate treatment and implement proper biosecurity measures to prevent the spread of disease. Moreover, the detection of emerging pathogens, such as CPnV and SARS-CoV-2, is essential to understand their epidemiology and, in the case of SARS-CoV-2 and influenza A virus A, to inform the owners and public health officials. Multiplex one-step qPCR or RT-qPCR assays allow the rapid identification of up to four targets in a single reaction. Therefore, this technique is now widely adopted in veterinary diagnostic laboratories [51,52,53,54]. Panels of qPCR/RT-qPCR and multiplex qPCR/RT-qPCR were previously developed for the detection of CIRDC-associated pathogens but they neither include all of the CIRDC-associated pathogens nor do they incorporate SARS-CoV-2 for its simultaneous detection [4,8,21]. In this study, the new qPCR/RT-qPCR panel developed for the detection of pathogens associated with CIRDC (including eight viruses and four bacteria), influenza A virus (H1N1, H3N2, and H3N8), and SARS-CoV-2 shows high analytical specificity and sensitivity for all targets tested.

With the expansion of next-generation sequencing in the past decade, pathogen sequence data have dramatically increased, leading to the discovery of new variants and strains. Additionally, pathogens, especially RNA viruses, are subject to point mutations leading to rapid evolution, as illustrated by the recent SARS-CoV-2 pandemic [68]. It is therefore important to validate and modify existing assays for the optimal detection of circulating strains. All assays in this study were developed to target most of the strains published on the GenBank nucleotide database and Influenza Research Database. In addition, degeneracy has been added to previously published assays to target circulating strains. The addition of one nucleotide degeneracy in the previously published CAdV-2_H probe (position 10: T→Y) [63] allows our assay to match in silico at 100% with all the CAdV-2 sequences available without affecting the specificity of the assay. Using online Influenza virus A databases, a similar approach was applied on the CIV_M reverse primer (position 1: T→Y) [62].

Different species within the *Mycoplasma* genus were isolated from dogs but only two of them, *M. cynos* and *M. canis,* were reported to induce respiratory disease [12,21,22,23]. In order to differentiate these two strains, the qPCR assays specific to *M. cynos* and *M. canis* developed by Tallmadge et al. [58] were evaluated. Although the *M. cynos* assay was specific for the *M. cynos* reference strain DNA, the amplification of both the *M. cynos* (27544 Rosendal, ATCC^®^) and *M. canis* (NR-3865, ATCC^®^) reference strain DNA was observed with the *M. canis* assay. For this reason, we developed a different *M. canis* qPCR assay targeting the elongation factor tuf gene (M.canis_tuf). This assay was specific for *M. canis* without the amplification of the *M. cynos* reference strain DNA. An in silico analysis was also performed for the development of new assays for the specific detection of CDV, CPiV, CPnV, CRCoV, CIV H3N2, and CIV H1N1 subtypes; all of those assays have shown an excellent specificity.

The analytical sensitivity of each multiplex qPCR and RT-qPCR assay was evaluated in this study and compared to singleplex assay formats. The nearly perfect linearity (R^2^ ≥ 0.997) and high amplification efficiency (>94%) demonstrate the high sensitivity of our panel of multiplex RT-qPCR assays for the detection of canine respiratory pathogens, without a loss of analytical sensitivity when used in multiplex conditions. Additionally, the detection of low genome copy numbers in clinical specimens is critical to the assay’s sensitivity. Here, a high analytical sensitivity was observed for most of our assays when used in multiplex with a LOD_95_ ≤ 15 copies/μL. While a lower sensitivity was observed for *B. bronchiseptica*, *M. canis,* and *M. cynos* (LOD_95_ ≤ 60 copies/μL), these results remained robust. Additionally, the excellent intra-run repeatability and inter-run reproducibility at high to low concentrations of target demonstrated that our panel of four multiplex qPCR/RT-qPCR assays ensure a high quality diagnostic and reproducibility of the results. In view of our data, we have demonstrated that using our assays in a multiplex format did not affect the analytical parameters. Additionally, these parameters were similar to those from other assays recently developed in our laboratory for the detection of canine enteric viruses and feline respiratory pathogens [69,70]. In this study, two to three log_10_ improvement of the LOD_95_ was determined when compared to the previously published multiplex PCR assays for the detection of CAdV-2, CDV, CIV, and CPiV [38]. However, the LOD_95_ determined in this study was in the same range of the recent three-panel triplex qPCR assays for the detection of nine pathogens associated with CIRDC (CAdV-2, CHV-1, CPiV, CDV, CIA, CRCoV, *M. cynos*, *M. canis,* and *B. bronchiseptica*) [21]. To our knowledge, no previous panel of multiplex qPCR/RT-qPCR assays was developed for the simultaneous detection of twelve canine respiratory pathogens and associated with the detection of SARS-CoV-2.

Our panel of qPCR/RT-qPCR was used to evaluate clinical samples collected from CIRDC-suspected dogs. While 36.8% of the samples were positive for only a single pathogen, 30.3% of the samples yielded positive results for at least two pathogens, demonstrating the high rate of co-infections, as previously reported [4,8,24,71]. This observation supports the need for a simultaneous detection of all the pathogens involved in CIRDC in order to inform treatment strategies. Among the positive samples, 82.4% were positive for *M. canis* or *M. cynos*, showing the common occurrence of these bacteria in CIRDC-suffering dogs, as recently highlighted in different studies [12,21]. As *M. canis* was detected in 73.9% of multi-infected dogs in this study, it is hypothesized that either this pathogen provides favorable conditions for secondary infections in the upper airways, or is a normal commensal of the upper respiratory tract in both health and disease, as previously suggested [23]. *B. bronchiseptica*, which is one of the most common pathogens found in CIRDC-suffering dogs [4,8,21], was surprisingly detected in only 2.6% dogs in our study. The low detection rate of *B. bronchiseptica* in the samples tested here can be explained by the lowest analytical sensitivity of this test (LOD_95_ = 43 copies/μL) when compared to the other targets.

The performance of the CRA_4 assay was not evaluated using field samples as no CIV-positive samples were identified during the study period. The emerging virus, CPnV, was also detected in two CIRD-suffering dogs located in the same shelter; co-infection with CRCoV and *M. cynos* was observed in one of the CPnV-infected dogs. SARS-CoV-2 was detected in four dogs in the absence of co-infection with other CIRDC pathogens. This result confirms that SARS-CoV-2 may induce respiratory disorders in dogs, highlighting the importance of including it in molecular diagnostic assays for CIRDC. Additionally, as the owners of two of these dogs were known to be positive for SARS-CoV-2 before their dogs, human-to-dog transmission was highly suspected. These results underline the need for the continued surveillance of SARS-CoV-2 infections in canine populations. In addition, the versatility of this panel allows the replacement or adjustment of SARS-CoV-2 detection for the identification of other emerging pathogens in the future.

## 5. Conclusions

To conclude, this study highlights the robustness of our new qPCR/RT-qPCR panel for the detection of SARS-CoV-2 and the eleven most important CIRDC-associated pathogens in clinical specimens. Therefore, this panel is suitable for routine diagnostics and the rapid identification of pathogens associated with CIRDC.

## Figures and Tables

**Table 1:** Panel of prototype canine respiratory viruses and bacteria, SARS-CoV-2 variants of concern, and other canine pathogens used to assess the specificity of each qPCR and RT-qPCR assay.

Pathogens	Reference Strain	Source
Canine Herpesvirus 1 (CHV-1)	VR-552^TM^	ATCC^®^
Canine Adenovirus 2 (CAdV-2)	VR-800^TM^	ATCC^®^
Canine Parainfluenza Virus (CPiV)	VR-399^TM^	ATCC^®^
Canine Respiratory Coronavirus (CRCoV)	VSL-1471	Cornell University^a^
Canine Distemper Virus (CDV) Lederle Avirulent	NR-3845	BEI Resources
Murine Pneumonia virus (MPV)	VR-1819	ATCC^®^
SARS-CoV-2 USA-WA1/2020	NR-52281	BEI Resources
SARS-CoV-2 Alpha (Lineage B.1.1.7)	NR-54020	BEI Resources
SARS-CoV-2 Beta (Lineage B.1.351)	NR-55282	BEI Resources
SARS-CoV-2 Delta (Lineage B.1.617.2)	NR-55671	BEI Resources
SARS-CoV-2 Omicron (Lineage B.1.1.529)	NR-56461	BEI Resources
Canine Influenza A (CIV) H3N2	VLS-1355	Cornell University ^a^
CIV H3N8	A/Ca/FL/15592/04	Cornell University ^b^
CIV H3N8	A/Ca/FL/61156.2/07	Cornell University ^b^
Influenza A virus, A/California/04/2009 (H1N1)pdm09	NR-13658	BEI Resources
*Bordetella bronchiseptica*, E014	NR-44164	BEI Resources
*Streptococcus equi* susb. *zooepidemicus* Farrow and Collins	700400^TM^	ATCC^®^
*Mycoplasma cynos* Rosendal	27544^TM^	ATCC^®^
*Mycoplasma canis*, PG 14	NR-3865	BEI Resources
Canine Adenovirus 1 (CAdV-1)	VR-293^TM^	ATCC^®^
Canine Enteric Coronavirus (CECoV), UCD1	NR-868	BEI Resources
*Mycoplasma felis* Cole et al.	23391^TM^	ATCC^®^

^a^ Kindly provided by Dr. Diego Diel; ^b^ Kindly provided by Dr. Edward Dubovi; ATCC^®^: American Type Culture Collection; LADDL: Louisiana Animal Disease Diagnostic Laboratory.

**Table 2:** Primers and probe sequences used for the detection of pathogens associated with CIRDC and SARS-CoV-2.

Target (Gene)	Oligonucleotide ID	Primers and Probe Sequences (5’–3’)	Nucleotide Position	Product Size(bp)	GenBank Accession	Reference
CPiV (Nucleoprotein)	CPiV_N-F CPiV_N-R CPiV_N-P	ACCATCAGCCACAATGCTCA	298–317		EF543648.1	This article
		AGCGGAATGATCCCTCCTCA	401–382	104		
		FAM-AGCTGACCAGTCACCAGAAGC-QSY	331–351			
Canine Influenza A virus (CIV) (Matrix protein)	CIV_M-F CIV_M-R1 ^a^ CIV_M-R2 ^a^ CIV_M-P	AGATGAGTCTTCTAACCGAGGTCG	24–47		MF173222.1	[62,64] with modification
		YGCAAAGACATCTTCAAGTCTCTG TGCAAAGACACTTTCCAGTCTCTG	124–101 124–101	101		
		VIC-TCAGGCCCCCTCAAAGCCGA-QSY	74–93			
CAdV-2 (Hexon)	CAdV2_H-F CAdV2_H-R CAdV2_H-P	AGTAATGGAAACCTAGGGG	17,821–17,839		U77082.1	[63] with modification
		TCTGTGTTTCTGTCTTGC	17,900–17,883	80		
		ABY-TCAGTCATCYCAGCTCAATGCCGTG-QSY	17,874–17,850			
CDV (Phosphoprotein)	CDV_P-F CDV_P-R CDV_P-P	ACTATTGAGAGACCTCCAGCTGAAA	1296–1320		AB028914.1	This article
		TGCGGTATCCTTCGGTTTGT	1374–1355	79		
		JUN-CCGATTGCCGAGCTAGACTCTTTGTCA-QSY	1352–1326			
SARS-CoV-2 (Nucleocapsid)	2019-nCoV_N1-F 2019-nCoV_N1-R 2019-nCoV_N1-P	GACCCCAAAATCAGCGAAAT	28,287–28,306		MN985325.1	[55]
		TCTGGTTACTGCCAGTTGAATCTG	28,358-28,335	72		
		FAM-ACCCCGCATTACGTTTGGTGGACC-QSY	28,309–28,332			
CPnV (Nucleocapsid)	CPnV_N-F CPnV_N-R CPnV_N-P	CAGGACAAGTTATGCTRAGGT	1825–1845		NC_025344.1	This article
		CTCAACCACCTGTTCCATCTC	1925–1905	101		
		VIC-AGCTTGAACACTAGCATGGCCTAGC-QSY	1904–1880			
CRCoV (Nucleocapsid)	CRCoV_N-F CRCoV_N-R CRCoV_N-P	CCTCTGGAAATCGTTCTGGTAA	8273–8294		DQ682406.1	This article
		GCTTGGGTTGAGCTCTTCTA	8371–8352	99		
		ABY-ACTGATCGGCCCACTTAAGGATGC-QSY	8320–8297			
CHV-1 (Glycoprotein B)	CHV_gB-F CHV_gB-R CHV_gB-P	ACAGAGTTGATTGATAGAAGAGGTATG	439–465		AF361073.1	[56]
		CTGGTGTATTAAACTTTGAAGGCTTTA	574–548	136		
		JUN-TCTCTGGGGTCTTCATCCTTATCAAATGCG-QSY	539–510			
*Bordetella bronchiseptica* (Intergenomic region between flaA and fliA B)	Fla2-F Fla12-R Fla-P	AGGCTCCCAAGAGAGAAAGGCTT	1,140,858–1,140,880	118	CP019934.1	[57]
		AAACCTGCCGTAATCCAGGC	1,140,975–1,140,956			
		FAM-ACCGGGCAGCTAGGCCGC-QSY	1,140,887–1,140,904			
*Mycoplasma cynos* (tuf)	Mcynos_tuf-F Mcynos_tuf-R Mcynos_tuf-P	TCTTCGTATTTAGCATCACCTTCAAGT	8234–8260		FJ896395.1	[58]
		TGATGGAGATAATGCGCCAAT	8305–8285	72		
		VIC-CTTTTAAAGCTGAACCACG-QSY	8262–8280			
*Streptococcus equi* subsp. *zooepidemicus* (sodA)	SodA-F SodA-R SodA-Bd 4116/06-R SodA-P	AGAGCAATTCACAGCAGCA	246–264		JN631988.1	[59]
		ACCAGCCTTATTCACAACCA ACCGGCTTGGTTAACCACTA	318–299 318–299	73		
		ABY-CAGGCCCAACCTGAGCCAAA-QSY	296–277			
*Mycoplasma canis* (tuf)	Mcanis_tuf-F Mcanis_tuf-R Mcanis_tuf-P	CAACAGCATCCATTAATTCCAT	305–326		FJ896394.1	This article
		ACGGATTTGACGGAGATAAC	412–393	108		
		JUN-TGAAGCTGATCCACGGATAATTGGAGC-QSY	366–392			
Canine H3N2 (Neuraminidase)	H3N2_NA-F H3N2_NA-R H3N2_NA-P	CCGTTGAAGGCAAAAGCTGT	1251–1270		MF173401.1	This article
		TCTCTTGTGGCCCTCCTCTT	1319–1300	69		
		FAM-AATAGGTGTTTTTATGTGGAGTTGAT-QSY	1274–1299			
Canine H3N8 (Hemagglutinin)	H3N8_HA3-F H3N8_HA3-R H3N8_HA3-P	TCACATGGACAGGTGTCACTCA	448–469		MF173285.1	[60,61]
		GGCTGATCCCCTTTTGCA	506–489	59		
		JUN-AACGGAAGAAGTGGAGC-QSY	471–487			
Canine H1N1 (Neuraminidase)	H1N1_NA-FH1N1_NA-R H1N1_NA-P	GCGGGCAATTCCTCTCTC	256–276		MG254090.1	This article
		CTTGGAACCGATTCKTACACTRT	333–311	78		
		ABY-TGYCCTGTTAGTGGATGGGCTATATACAGT-QSY	274–303			

ABY, ABY^TM^ dye; F: forward primer; FAM, 6-carboxyfluorescein dye; JUN, JUN™ dye; P: probe; QSY, QSY™ quencher; R: reverse primer; VIC, VIC™ dye; ^a^ CIV_M-R1 and CIV_M-R2 were used at equimolar amount (200 nM).

**Table 3:** Analytical performance of singleplex and four-plex qPCR/RT-qPCR assays for the detection of pathogens associated with CIRDC and SARS-CoV-2.

Assay	Target	Parameter	Slope	Linearity (R^2^)	Efficiency (%)	LOD_95%_ (Copies/µL)	Detection Rate Limit (Copies/µL)	Ct Cut-Off
CRA_1	CAdV-2 (H)	Singleplex	−3.024	0.999	114.14	4	10	34
		Four-plex	−3.057	0.998	112.38	5	10	35
	CPiV (NP)	Singleplex	−3.037	0.999	113.44	5	10	35
		Four-plex	−3.053	0.999	112.59	5	10	34
	CDV (P)	Singleplex	−3.042	0.999	113.17	4	10	36
		Four-plex	−3.090	0.999	110.68	4	10	35
	CIA (M)	Singleplex	−3.142	0.999	108.10	4	10	37
		Four-plex	−3.113	0.999	109.52	4	10	34
CRA_2	CRCoV (N)	Singleplex	−3.258	0.999	102.74	10	100	35
		Four-plex	−3.335	0.999	99.46	8	10	34
	SARS-CoV-2 (N1)	Singleplex	−3.248	0.999	103.18	5	10	39
		four-plex	−3.313	0.999	100.37	5	10	37
	CHV-1 (gB)	Singleplex	−3.264	0.999	102.48	12	100	35
		Four-plex	−3.350	0.999	98.84	14	100	38
	CPnV (N)	Singleplex	−3.309	0.999	100.54	6	10	40
		Four-plex	−3.379	0.996	97.67	6	10	36
CRA_3	*S. equi* subsp. *zooepidemicus* (sodA)	Singleplex	−3.240	0.999	103.54	6	10	34
		Four-plex	−3.169	0.998	106.80	15	100	35
	*B. bronchiseptica* (flaA-fliA-B)	Singleplex	−3.229	0.999	104.03	6	10	35
		Four-plex	−3.150	0.999	107.71	43	100	40
	*M. canis* (tuf)	Singleplex	−3.222	0.999	104.35	28	100	35
		Four-plex	−3.129	0.999	108.73	60	100	31
	*M. cynos* (tuf)	Singleplex	−3.266	0.999	102.39	43	100	38
		Four-plex	−3.210	0.998	104.89	53	100	35
CRA_4	CIA - H3N2 (NA)	Singleplex	−3.345	0.999	99.05	6	10	37
		Four-plex	−3.324	0.998	99.91	8	10	34
	CIA - H1N1 (NA)	Singleplex	−3.262	0.998	102.56	5	10	35
		Four-plex	−3.266	0.998	102.39	12	100	32
	CIA - H3N8 (HA)	Singleplex	−3.472	0.997	94.10	8	10	39
		Four-plex	−3.418	0.997	96.14	8	10	35
	CIA (M)	Singleplex	−3.398	0.999	97.28	6	10	39
		Four-plex	−3.379	0.999	97.67	8	10	38

CRA: canine respiratory assay; R^2^: linearity; LOD_95%_ limit of detection 95%; Ct: cycle threshold.

**Table 4:** Precision assessment of each four-plex qPCR and RT-qPCR assay.

Assay	Target	Intra-Run Variability CV (%) ^#^			Inter-Run Variability CV (%) ^#^		
		10^5^ Copies/µL	10^4^ Copies/µL	10^3^ Copies/µL	10^5^ Copies/µL	10^4^ Copies/µL	10^3^ Copies/µL
CRA_1	CAdV-2 (H)	0.55	2.58	2.00	1.19	0.83	1.92
	CPiV (NP)	0.56	2.33	0.95	1.44	0.65	0.78
	CDV (P)	1.01	2.50	3.05	1.60	1.65	2.56
	CIA (M)	0.65	2.27	0.69	1.69	0.57	0.35
CRA_2	CRCoV (N)	1.09	1.19	2.66	0.88	1.05	1.72
	SARS-CoV-2 (N1)	0.84	1.14	1.79	0.85	0.73	1.13
	CHV-1 (gB)	1.66	3.96	2.95	1.58	1.61	3.80
	CPnV (N)	0.66	0.89	2.49	1.77	1.57	1.79
CRA_3	*S. equi* subsp. *zooepidemicus* (sodA)	0.82	2.29	3.66	1.00	2.67	3.07
	*B. bronchiseptica* (flaA–fliA-B)	0.43	1.86	2.96	0.57	1.14	4.83
	*M. canis* (tuf)	1.29	3.23	5.70	1.66	2.68	4.23
	*M. cynos* (tuf)	0.57	1.53	3.54	0.96	2.06	2.85
CRA_4	CIA-H3N2 (NA)	0.82	0.78	1.09	0.50	0.60	3.07
	CIA-H1N1 (NA)	0.67	0.58	1.29	0.90	1.48	1.49
	CIA-H3N8 (HA)	1.42	1.80	3.93	1.20	1.42	4.19
	CIA (M)	0.28	0.43	0.86	1.23	1.45	2.67

^#^ CV (%): Coefficient of variation = (standard deviation of replicates [log_10_ copies/µL] ÷ Average of replicates [log_10_ copies/µL]) × 100.

## Data Availability

The data presented in this study are available on request from the corresponding author.

## Supplementary Materials

The following supporting information can be downloaded at: https://www.mdpi.com/article/10.3390/v15091881/s1, Figure S1: Assessment of the specificity of all qPCR and RT-qPCR assays using reference strain DNA/RNA; Figure S2: analytical sensitivity determination of singleplex and multiplex qPCR and RT-qPCR assays using plasmid DNA and *IVT* RNA; Table S1: origin of the samples used in this study; Table S2: detection rate of CIRDC-associated pathogens and SARS-CoV-2 in clinical specimens using the newly developed panel; Table S3: detection rate of single agent infections and co-infections associated with CIRDC in the clinical samples tested using the newly developed panel.
