# Supplementary material for: Development and Validation of a Panel of One-Step Four-Plex qPCR/RT-qPCR Assays for Simultaneous Detection of SARS-CoV-2 and Other Pathogens Associated with Canine Infectious Respiratory Disease Complex
Source: Viruses. 2023 Sep 5;15(9):1881. doi: 10.3390/v15091881 (PMC10535912; doi:10.3390/v15091881)
Supplement: Supplementary file 1 [file viruses-15-01881-s001.zip › viruses-2572311-supplementary.pdf]

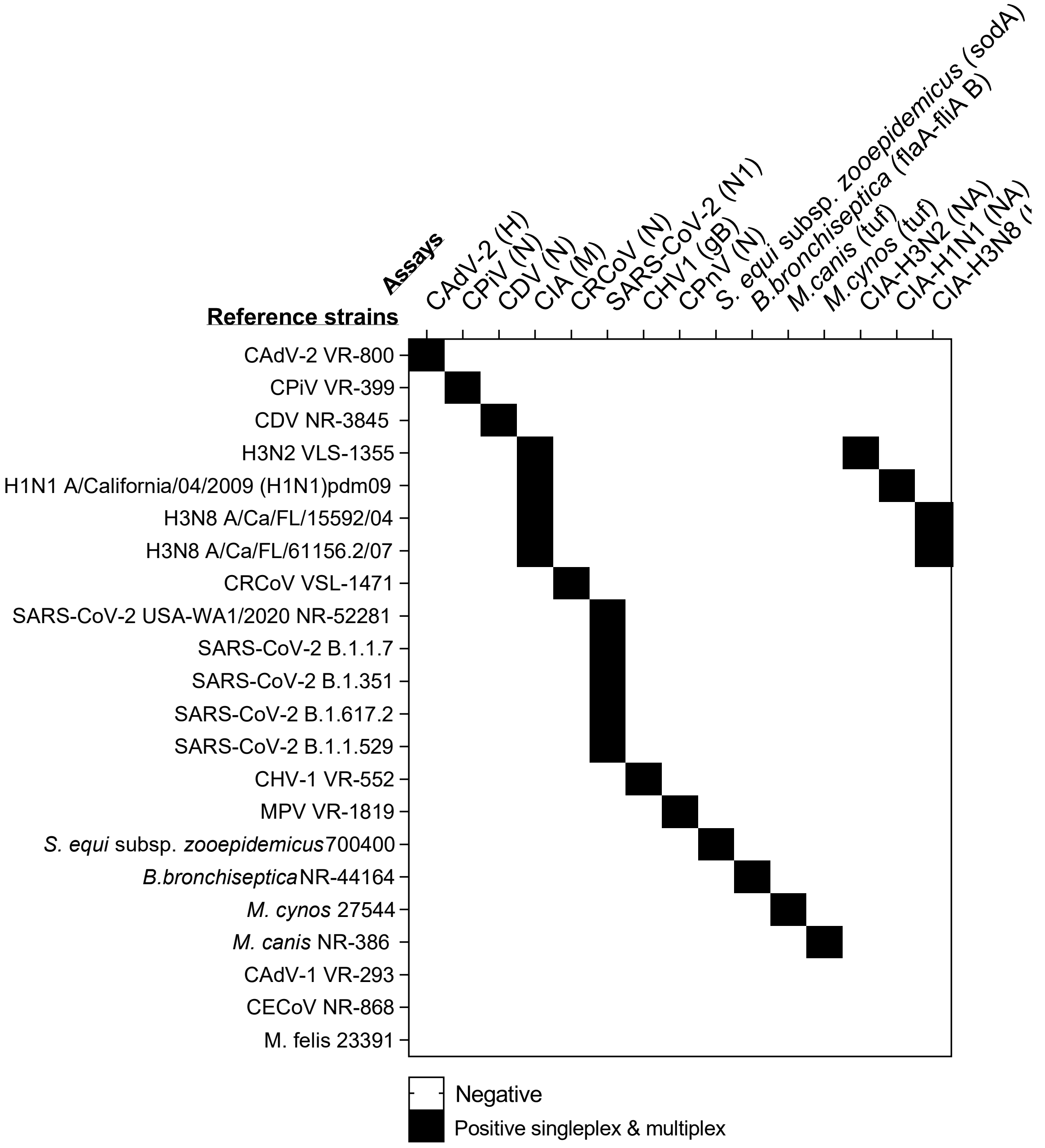

Figure S1. Assessment of the specificity of all qPCR and RT-qPCR assays using reference strain DNA/RNA. Each column corresponds to one specific qPCR/RT-qPCR assay and each row corresponds to one specific reference strain of virus or bacteria. Specificity was assessed for each assay in singleplex and in multiplex. White cases correspond to the absence of detection while black cases correspond to DNA/RNA amplification in both singleplex and multiplex assays.

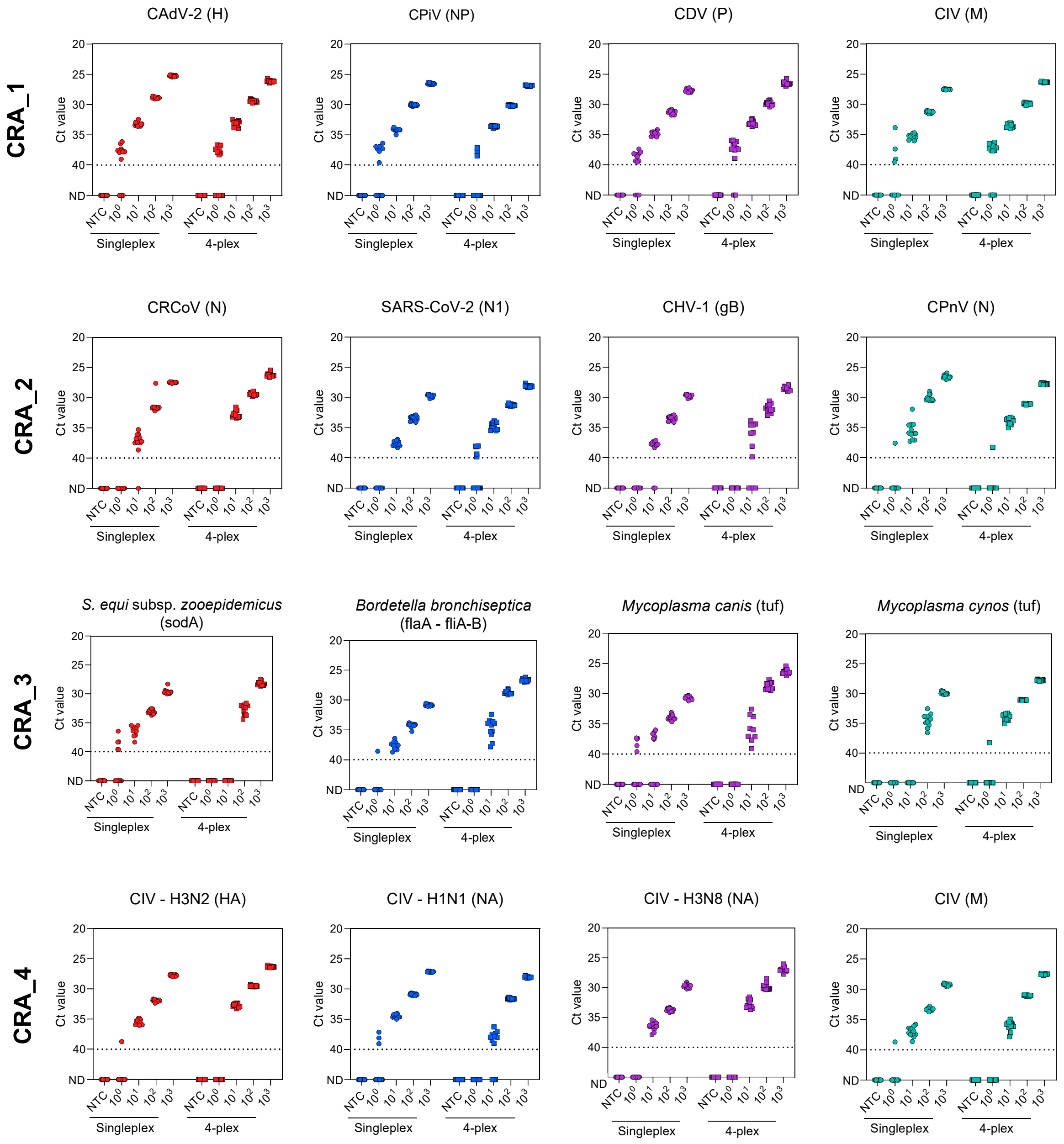

Figure S2. Analytical sensitivity determination of singleplex and multiplex qPCR and RT-qPCR assays using plasmid DNA and *IVT* RNA. Each assay was performed using 12 replicates ranging from 10^3 to 10^0 copies/μl. Each circle and square indicate the Ct value of one replicate obtained by singleplex and multiplex amplification, respectively. Short solid lines indicate the median Ct value and dashed lines indicate the detection limit. ND: not detected; NTC: no template control.

Table S1. Origin of the samples used in this study. Two pools of tissues, 45 nasal swabs and 29 pharyngeal swabs were collected from 50 CIRDC-suspected dogs during the period between 2020 and 2023.

Location	Dogs	Tissues pool (n=2)	Nasal swab (n=45)	Pharyngeal swab (n=29)
LADDL	1	X		
	2		X	X
	3		X	X
	4		X	X
	5		X	X
	6		X	X
	7		X	X
	8		X	X
	9		X	X
	10		X	X
	11		X	
	12			X
	13			X
	14		X	
	15			X
	16		X	
	17		X	
	18		X	
	19		X	
	20		X	
	21		X	
	22		X	
	23	X		
Shelter 1	24		X	X
	25		X	X
	26		X	X

	27	X	X
	28	X	X
	29	X	X
	30	X	X
	31	X	X
	32	X	X
	33	X	X
	34	X	X
	35	X	X
	36	X	X
	37	X	X
	38	X	
	39	X	X
	40	X	X
	41	X	X
Shelter 2	42	X	
	43	X	
	44	X	
Shelter 3	45	X	
	46	X	
	47	X	
	48	X	
	49	X	
	50	X	

Table S2. Detection rate of CIRDC-associated pathogens and SARS-CoV-2 in clinical specimens using the newly developed panel.

**Pathogens**	**No. of positive samples (n = 51/76)***	**% positive samples (67.1%)***
CAdV-2	3	3.9
CPiV	3	3.9
CDV	3	3.9
CIV	0	0
CRCoV	15	19.7
SARS-CoV-2	4	5.3
CHV-1	6	7.9
CPnV	4	5.3
S. equi subsp. *zooepidemicus*	0	0
B. bronchiseptica	2	2.6
M. canis	23	30.3
M. cynos	19	25.0

*Positive samples for at least one pathogen

Table S3. Detection rate of single agent infections and co-infections associated with CIRDC in the clinical samples tested using the newly developed panel.

**Pathogens**	**No. of Positive samples**	**% of Positive samples**
Respiratory infections associated with one pathogen	***28/76***	***36.8***
CPnV	2	2.6
CDV	3	3.9
CHV-1	3	3.9
CRCoV	4	5.3
SARS-CoV-2	4	5.3
M. cynos	6	7.9
M. canis	6	7.9
Respiratory infections associated with two pathogens	***16/76***	***21.1***
CPiV + *B. bronchiseptica*	1	1.3
CHV-1 + *M. cynos*	1	1.3
CAdV-2 + *M. canis*	1	1.3
M. cynos + *M. canis*	6	7.9
CRCoV + *M. canis*	7	9.2
Respiratory infections associated with three pathogens	***6/76***	***7.9***
CPiV + CHV-1 + *M. cynos*	1	1.3
CPiV + CAdV-2 + *M. canis*	1	1.3
CAdV-2 + *M. cynos* + *M. canis*	1	1.3
CRCoV + *M. cynos* + *M. canis*	1	1.3
CRCoV + CPnV + *M. cynos*	2	2.6
Respiratory infections associated with four pathogens	***1/76***	***1.3***
CRCoV + CHV-1 + *B. bronchiseptica* + *M. cynos*	1	1.3

*15/76 (32.9%) specimens were negative for the pathogens tested
